# Glycosylation as a Main Regulator of Growth and Death Factor Receptors Signaling

**DOI:** 10.3390/ijms19020580

**Published:** 2018-02-15

**Authors:** Inês Gomes Ferreira, Michela Pucci, Giulia Venturi, Nadia Malagolini, Mariella Chiricolo, Fabio Dall’Olio

**Affiliations:** Department of Experimental, Diagnostic and Specialty Medicine (DIMES), General Pathology Building, University of Bologna, 40126 Bologna, Italy; ines.gomesferreira@unibo.it (I.G.F.); michela.pucci3@unibo.it (M.P.); giulia.venturi13@unibo.it (G.V.); nadia.malagolini@unibo.it (N.M.); mariella.chiricolo@unibo.it (M.C.)

**Keywords:** cancer, growth factor receptors, glycosylation, galectins, gangliosides, signal transduction

## Abstract

Glycosylation is a very frequent and functionally important post-translational protein modification that undergoes profound changes in cancer. Growth and death factor receptors and plasma membrane glycoproteins, which upon activation by extracellular ligands trigger a signal transduction cascade, are targets of several molecular anti-cancer drugs. In this review, we provide a thorough picture of the mechanisms bywhich glycosylation affects the activity of growth and death factor receptors in normal and pathological conditions. Glycosylation affects receptor activity through three non-mutually exclusive basic mechanisms: (1) by directly regulating intracellular transport, ligand binding, oligomerization and signaling of receptors; (2) through the binding of receptor carbohydrate structures to galectins, forming a lattice thatregulates receptor turnover on the plasma membrane; and (3) by receptor interaction with gangliosides inside membrane microdomains. Some carbohydrate chains, for example core fucose and β1,6-branching, exert a stimulatory effect on all receptors, while other structures exert opposite effects on different receptors or in different cellular contexts. In light of the crucial role played by glycosylation in the regulation of receptor activity, the development of next-generation drugs targeting glyco-epitopes of growth factor receptors should be considered a therapeutically interesting goal.

## 1. Introduction

Cells receive the order to proliferate, survive, or die usually from the outside, in the form of small proteins that bind to specific receptors on their surface. Binding of these ligands, which include growth factors, cytokines, hormones, and death-inducing molecules, to their receptors triggers intracellular signaling cascades, which ultimately lead to gene transcription. Most of these receptors are classical tyrosine kinase receptors (RTKs). They are homo- or heterodimers in which the ligand binding triggers receptor dimerization and the autophosphorylation of tyrosine residues in the cytoplasmic portion, generating a transduction signal. The activity of a specific receptor, its turnover, and its ability to interact with adaptor molecules are finely regulated by post-translational mechanisms, including glycosylation [1,2,3]. In this review, after a brief outline of glycosylation, we will address the different mechanisms bywhich glycosylation modulates receptor activity. Notch receptors, which exhibit a very peculiar kind of glycosylation, have been the topic of recent reviews [4,5,6] and are not discussed here.

## 2. Outline of *N*- and *O*-Glycosylation

Glycoconjugates, which include glycoproteins, proteoglycans, and glycolipids, are biological molecules comprised of a sugar portion linked to proteins or lipids. The two major types of protein- linked sugar chains in glycoproteins are referred to as *N*-linked and *O*-linked. In the first, the sugar moiety is linked through a bond between the reducing *N*-acetylglucosamine (GlcNAc) residue and the nitrogen of asparagine; in the second, through a link between the reducing *N*-acetylgalactosamine (GalNAc) residue and the hydroxyl groups of serine or threonine. Other types of sugar–protein structures, such as *O*-GlcNAc, *O*-mannose, or *O*-fucose, play specific roles but their discussion is beyond the scope of this review. The biosynthesis of the sugar portion of glycoconjugates is mediated by glycosyltransferases. These enzymes transfer monosaccharides from a donor substrate (usually a sugar-nucleotide, such as UDP-GlcNAc or CMP-sialic acid) to an acceptor, which can be a nascent sugar chain. The biosynthesis of *N*- and *O*-linked chains follows completely different pathways. *N*-linked chains are synthesized as a pre-assembled dolichol-linked glycan comprised of twoGlcNAc, nine mannose (Man), and three glucose (Glc) residues in the rough endoplasmic reticulum, which arethen transferred en bloc to an asparagine residue of the nascent protein [7]. A necessary but not sufficient consensus motif for *N*-glycosylation is Asn-X-Ser/Thr, where X is any aminoacid except proline. Successively, the threeGlc and a total of six Man residues are trimmed during transit along the exocytic pathway and replaced with GlcNAc, galactose (Gal), sialic acid (Sia), and fucose (Fuc) residues, arranged in a variety of structures expressed on several branches (*antennae*), whose number usually ranges from two to four. On the contrary, the biosynthesis of *O*-linked chains proceeds from the stepwise addition of single monosaccharides along the exocytic pathway. The addition of the first GalNAc residue to the peptide can be mediated by 20 different peptide:GalNac transferases with subtle substrate differences [8] (see Table 1 for a list of glycosyltransferases mentioned in this review and their cognate carbohydrate structures). Given their ubiquitous presence and abundance in all cellular compartments, extracellular spaces, and body fluids, glycans exert their biological effects by modulating functions of proteins and lipids to which they are attached, acting as a “fine tuning” of their biological activities [9]. In cancer, glycosylation undergoes profound changes (reviewed in [10,11]) thataffect a variety of membrane proteins, including growth factor receptors. Altered glycosylation of growth factor receptors is partly responsible for their deranged activity and the cancer cell phenotype.

## 3. General Mechanisms by Which Glycosylation Affects Receptor Activity

The mechanisms by which glycosylation of membrane receptors affects their activity, modulating the flow of information from the plasma membrane to the nucleus (outside-in) and eventually transcription activity [10,94], can be classified in the following three broad conceptual groups. It should be emphasized that the three mechanisms outlined below are not mutually exclusive.

### 3.1. Direct Effect of Glycosylation

Whole sugar chains, such as *N*-glycans or specific carbohydrate structures, affect intracellular transport, ligand binding, oligomerization and signaling of the receptors [15,95]. Using *N*-glycosylation inhibitors, such as tunicamycin, or *N*-glycosylation sites mutagenesis it has been possible to assess the role played by *N*-glycans as a whole. For instance, it has long been known that the use of the inhibitor tunicamycin to prevent *N*-glycosylation can negatively affect the initial folding of ER-synthesized proteins. On the other hand, the role played by specific sugar epitopes, for example core fucose or the Lewis antigens (Table 1), is indicated by studies in which specific glycosyltransferases have been genetically manipulated in cell lines [96] or mice [28].

### 3.2. Galectin Binding

**Figure 1:**
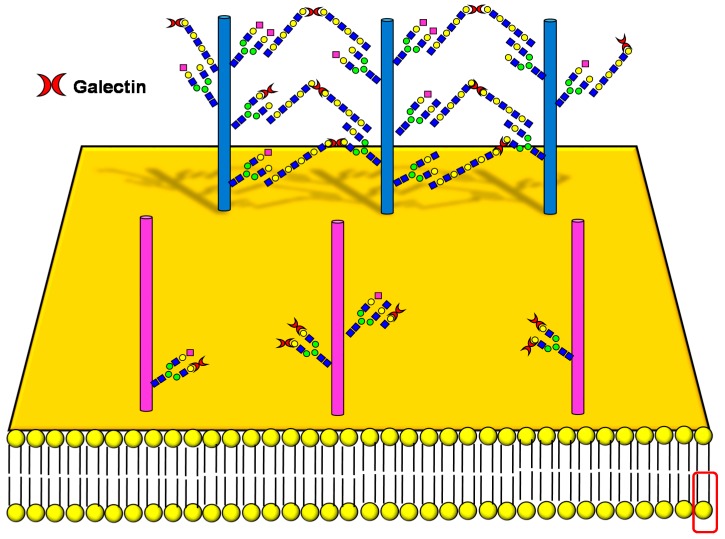
Schematic representation of a plasma membrane area with two different generic growth factor receptors. In light blue is represented a receptor with a high number of *N*-linked chains bearing the β1,6-branched polylactosaminic chains, which are ideal ligands for galectins (in red). The binding of galectins to polylactosaminic chains results in a lattice, which stabilizes receptors on the cell membrane. In pink is represented a receptor with a small number of *N*-linked chains with a few β1,6-branching and no polylactosaminic chains. This type of glycosylation makes galectins unable to form a lattice and stabilize receptors. Sugar codes are as in the legend of Table 1.

Galectins, extracellular multivalent lectins [97], bind to the carbohydrate structures of the receptors forming a lattice (Figure 1), which regulates their turnover on the plasma membrane [98], usually potentiating the signaling downstream [99]. The ability of receptors to bind galectins is critically dependent on the degree of branching of their *N*-linked chains (Figure 1), in particular on the presence of the β1,6-branch synthesized by GlcNAc transferase-V (GnT-V) encoded by the *MGAT5* gene (Table 1). A complex mechanism links β1,6-branching, Golgi concentration of UDP-GlcNAc (the donor substrate of GlcNAc transferases) and the turnover of receptors on the membrane [100]. In fact, high glucose concentration results in increased Golgi levels of UDP-GlcNAc and MGAT5 activity, which, in turn, stimulate β1,6-branching, leading to the biosynthesis of binding sites for galectin-3. Interaction of receptors with galectin-3 and their consequent entrapment in a lattice regulates their permanence on the cell membrane [101]. It has been shown that growth-promoting receptors (e.g., the receptors of epidermal growth factor, EGFR; of insulin like growth factor, IGFR; of fibroblast growth factor, FGFR) exhibit a higher number of *N*-linked chains than inhibitory receptors (e.g., transforming growth factor-β receptor, TGFBR and cytotoxic T-lymphocyte antigen 4, CTLA-4) [100,102]. The increase of intracellular glucose concentration generates a hyperbolic activation profile for receptors with a high number of *N*-glycans but a sigmoid profile for those with a lower number. This makes inhibitory receptors much more sensitive to increasing glucose concentration than growth-promoting receptors, resulting in their switch-like responses (Figure 1) [100,102].

### 3.3. Interaction with Gangliosides

**Figure 2:**
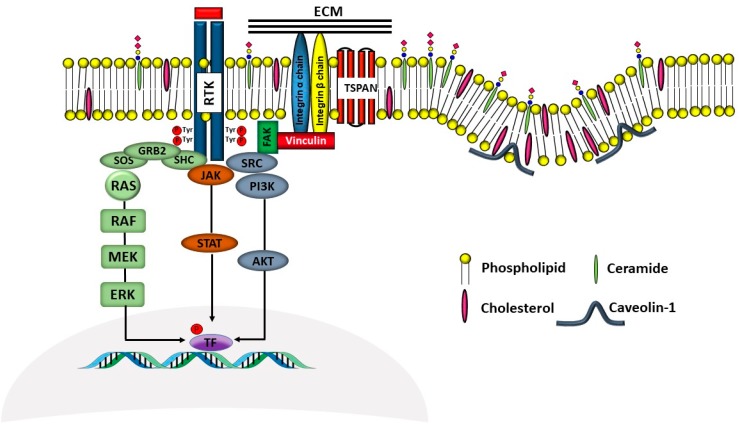
Schematic representation of membrane microdomains. The complex interactions among RTKs, integrins and tetraspanins with signal transduction molecules leading to gene transcription, are depicted in a simplified representation. The left part of the Figure represents a tetraspanin microdomain, while the right part represents a caveola with caveolin-1. Sugar codes are as in the legend of Table 1.

Gangliosides are sialic acid containing glycolipids (Table 1) that can modulate receptor activity because they colocalize with growth factor receptors and a variety of other molecules in specialized areas of the plasma membrane known as microdomains (Figure 2) [103,104]. Lipid rafts and tetraspanin-enriched microdomains are distinct structures. In both of them, signals coming from growth factor receptors are integrated with those coming from gangliosides and other microdomain components and conveyed to the nucleus by signal transduction pathways [105]. Glycosylation can regulate this system at the level of membrane receptors, gangliosides, or other microdomain components, such as cell adhesion molecules. Glycosylation of these components results from the activity of glycosyltransferases and glycosidases. A remarkably important glycosidase family is the NEU family of sialidases that embraces four enzymes (NEU1-4), which cleave sialic acids from glycoconjugates in different cellular localizations [106,107].

## 4. How Glycosylation Modulates the Activity of Specific Receptors

### 4.1. Receptors of the ERBB Family

The ERBB family of RTK consists of four members (ERBB1-4), of which ERBB1 is also known as the EGF receptor (EGFR), while ERBB2 as HER2. Receptor homo- or hetero- dimerization induced by ligand binding triggers intracellular signaling, mainly through mitogen-activated protein kinase (MAPK), Janus kinase (JAK)/signal transducer and activator of transcription (STAT) and phosphatidylinositol 3-kinase (PI3K)/protein kinase B (Akt) pathways [108]. EGFR is also present in the nucleus of carcinoma cells, where it transcriptionally regulates several genes, including *CCND1* encoding cyclin D1 [109]. Overexpression, increased activity or mutations of EGFR are described in various human epithelial tumors, indicating its causative role in the etiology of human epithelial cancers. Thus, targeting of EGFR has been actively pursued over the last three decades as a treatment strategy. From these efforts, two fundamental approaches have proven to be useful. One approach involves the use of small molecule tyrosine kinase inhibitors such as Erlotinib (Tarceva) and Gefitinib (Iressa), both EGFR-specific tyrosine kinase inhibitors, while a second approach uses monoclonal antibodies (mAbs) such as Cetuximab (Erbitux), which prevent both ligand binding and dimerization with other HER family members. On the other hand, HER2 is the target of the monoclonal antibody Trastuzumab (Herceptin). These molecular drugs are in clinical use for the treatment of several cancers.

#### 4.1.1. Direct Effect of Glycosylation on ERBB Activity

EGFR has 11 typical (N-X-S/T) and 4 atypical (N-X-C) *N*-glycosylation consensus sequences and *N*-glycans account for about 40 kDa of its total molecular mass [2]. The number of potential *N*-glycosylation sites of the other ERBB members ranges from 8 of ERBB2 to 11 of ERBB4 [2]. As shown by site directed mutagenesis, some of the several *N*-glycans exposed on the extracellular region of ERBB receptors can directly modulate their biological activity, probably by preventing ligand-independent dimerization [12,13,14,110] and modulating intracellular transport [21].

Core fucose is required for the ligand binding ability [24] and intracellular signaling of EGFR [23]. In fact, it has been reported that, in the absence of core fucosylation, the biological function of the receptor may decrease and the process of hepatic carcinogenesis is inhibited [25]. Bisecting GlcNAc inhibits EGFR and integrin signaling through the Ras/MAPK pathway [30]. Among Lewis antigens, Lewis^y^(Le^y^) is the product of two fucosylation reactions: the addition of Fuc α1,2 linked to Gal and of Fuc α1,3 linked to GlcNAc (Table 1). The first reaction is catalyzed by FUT1 or FUT2, the second by FUT4. Genetic manipulation studies of these fucosyltransferase genes demonstrate the importance of Le^y^ for ERBB receptors activity and cell malignancy [50,51,52,53,54,55]. On the other hand, the presence of sialylated/fucosylated structures, presumably the sialyl Le^x^ (sLe^x^), on *N*-linked chains of EGFR reduces its ligand-induced dimerization and intracellular signaling, resulting in decreased invasion in lung cancer [46]. By contrast, expression of ERBB2 correlates with the expression of FUT3, the main fucosyltransferase responsible for the biosynthesis of the related antigen sialyl Le^a^ (sLe^a^) (Table 1) in gastric cancer specimens. Indeed, experiments of antigen blocking by anti sLe^a^ antibody in a gastric cancer cell line result in a dramatic downregulation of the ERBB2 protein [58].

Both α2,3 and/or α2,6 sialylation of EGFR reduce ligand binding and tyrosine phosphorylation [46] and increase Gefitinib sensitivity in Gefitinib-resistant cells [47]. The inverse correlation between EGFR signaling and sialylation (in this case, α2,6 sialylation mediated by ST6GAL1) was confirmed in a colon cancer cell model, although in this case reduced sialylation was associated with increased Gefitinib sensitivity [48]. Other members of the ERBB family appear to be negatively regulated by ST6GAL1 expression, albeit through an indirect and complex mechanism. In fact, reduction of ST6GAL1 expression, due to overexpression of mi-R199a, leads to a reduced protein level of the nectin-like Molecule 2/Cell Adhesion Molecule 1, which acts as an inhibitor of the ERBB2/ERBB3 signaling [111]. A further confirmation of the inverse effect of sialylation on EGFR activity comes from a study showing the activation of EGFR signaling, as a consequence of plasma membrane sialidase NEU3 overexpression [112,113]. However, an inhibitory rather than an activatory effect on EGFR signaling has been reported for NEU1 overexpression in airway cells [114]. The *O*-glycosylation of EGFR, mediated by GALNT2 or GALNT6, two of the 20 GalNAc transferases catalyzing the first step in *O*-linked biosynthesis (Table 1), results in enhanced malignancy of oral [64] and ovarian cancer [65], respectively. On the other hand, GALNT2 modification of EGFR reduces malignancy of hepatocarcinoma [66].

The β1,6-branched glycans and their biosynthetic enzyme MGAT5 play a very important role in enhancing EGFR activity. These mechanisms include the activation of p21-activated kinase 1, which leads to resistance to anoikis (a type of cell death triggered by the lack of adhesion to solid substrates) [34], the inhibition of the protein tyrosine phosphatase kappa (RPTPκ) (phosphatases usually counteract the activity of kinases, downregulating signaling) [35], and radioresistance [39], all related to the EGFR signaling pathway [38]. Knockdown of *Mgat5* inhibits EGFR internalization and intracellular signaling [37] and reduces dephosphorylation of focal adhesion kinase [36].

#### 4.1.2. Effect of Galectin Binding on ERBB Activity

As previously mentioned, the presence of β1,6-branching promotes a galectin lattice formation (Figure 1), which regulates the permanence of receptors on the plasma membrane [100]. However, caveolin-1 can antagonize galectin lattice formation, in particular when the degree of β1,6-branching is relatively low. In fact, when lattice formation is poor, EGFR localizes in caveolin-1 microdomains (Figure 2) where downstream signaling is inhibited [115]. MUC1 is a heavily *N*- and *O*-glycosylated membrane glycoprotein composed of two subunits: the extracellular *N*-terminal and the membrane C-terminal domain joined by a non-covalent link [116]. In cancer cells, the transmembrane portion of MUC1 and EGFR physically interact [117], although in normal epithelial cells this interaction is prevented by their differential localization at the apical and basolateral cell side, respectively. The phosphorylation at the C-terminal of MUC1 cytoplasmic portion is mediated by EGFR and regulates its interaction with p53 and β-catenin, which is necessary for gene transcription [118]. Galectin-3 plays a key role in the regulation of the intracellular trafficking and activation of MUC1 and EGFR [119,120,121]. The “Yamanaka factors” are a group of genes, including c-Myc, Sox2 and Oct4, whose expression generates “induced pluripotent stem cells” (iPSC) [122]. Galectin-3 promotes a self-fueling loop generating cancer stem cells (CSCs) through stimulation of EGFR, which, in turn, leads to increased c-Myc protein stability and Sox2 transcription. Oct4, in turn, promotes galectin-3 expression, enhancing a positive regulatory loop in lung CSCs [123]. In prostate cancer, overexpression of galectin-4 relates to malignancy because it binds on the *O*-linked carbohydrate antigen T (Table 1), activating EGFR/ERBB2 signaling [124]. On the other hand, a positive relationship among the expression of ST3GAL1, the enzyme thatconverts the T antigen in sialyl-T antigen (Table 1), EGFR signaling and malignancy has also been documented [71].

#### 4.1.3. Interaction of Gangliosides with ERBB Activity

**Figure 3:**
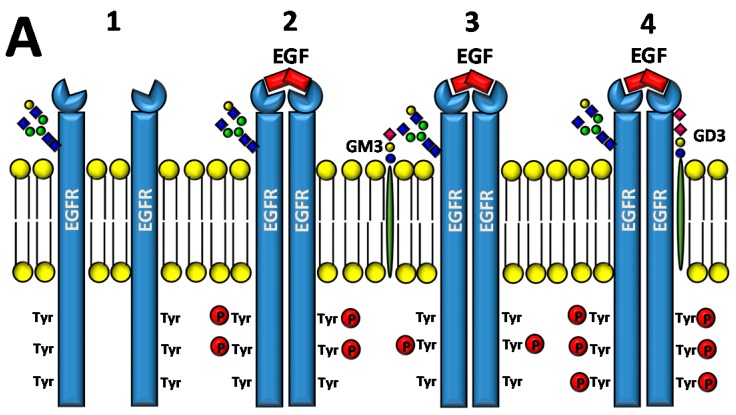

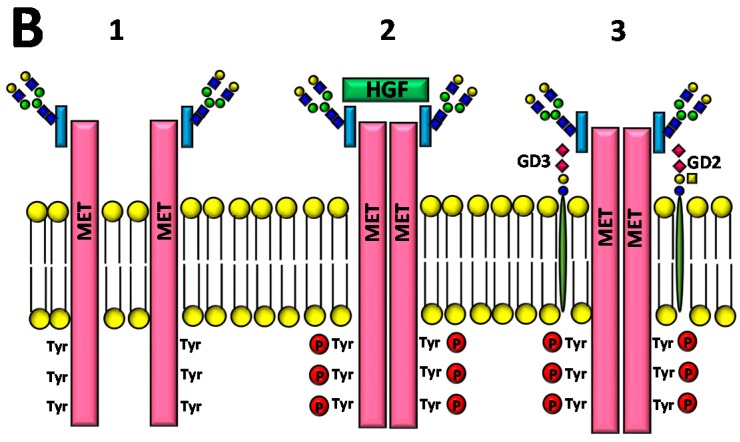
Effect of gangliosides on RTK activity. (**A**) Two EGFR monomers (1), in the presence of EGF, dimerize resulting in phosphorylation of tyrosine residues at their cytoplasmic portion (2). The interaction of the GlcNAc termini of the EGFR *N*-linked chains with ganglioside GM3 inhibits signaling (3), while interaction with GD3 reinforces it (4). (**B**) The MET receptor is comprised of a heavily glycosylated extracellular α chain (in blue) and a transmembrane β chain (in red), which undergoes phosphorylation of tyrosine residues of its cytoplasmic domain upon HGF binding (1 and 2). MET activation can be induced by interaction with disialogangliosides, such as GD3 and GD2, even in the absence of HGF (3). Sugar codes are as in the legend of Table 1.

Ganglioside regulation of ERBB receptors activity is crucially dependent on the number of gangliosides actually residing in microdomains (Figure 2). Several studies have provided information on the role of GM3 in the localization of ERBB receptors in relation to their phosphorylation [125,126,127,128]. Very recently, it has been reported that disialogangliosides, such as GD3 and GD2, enhance EGFR signaling, resulting in the expression of a cancer stem cell phenotype and a reduced sensitivity to Gefitinib [86]. In tetraspanin-enriched microdomains, various membrane receptors are organized by members of the tetraspanin superfamily. The importance of gangliosides in this structure is demonstrated by the fact that inhibition of their biosynthesis and expression of neuraminidase NEU3 results in altered association of EGFR with microdomains [129]. A complex interplay among integrins, caveolin, tetraspanin CD82 and gangliosides is at the basis of the modulation of EGFR activity by urokinase-type plasminogen activator (uPA) [130,131,132]. The interaction of the GlcNAc termini of the *N*-linked chains of EGFR with the sialylatedlactosaminic chain of the ganglioside GM3 (Figure 3A) provides an example of carbohydrate–carbohydrate interaction, which leads to downregulation of EGFR tyrosine kinase activity [76,77,78,79,80]. Interestingly, the cell surface β-galactosyltransferase-1 (B4GALT1), which behaves like a lectin, has also been reported to bind to EGFR (putatively through its GlcNAc termini), reducing its activation [6,133]. GM3 is the precursor of higher gangliosides. In fibroblasts from patients affected by an inactivating mutation of the GM3 synthase ST3GAL5 (Table 1), the ganglioside content (not only GM3) is 93% reduced. In these cells, the number of EGFR molecules is normal although the binding of EGF and the downstream signaling are reduced [134]. Probably, this effect is a balance between the loss of the inhibitory activity of GM3 and the loss of the stimulatory activity played by more complex gangliosides, such as GD1a [75,90,91].

### 4.2. Receptor of the Hepatocyte Growth Factor

The hepatocyte growth factor receptor (also known as c-Met), product of the *MET* gene, is expressed on epithelial cells of many organs and is composed by a highly glycosylated extracellular α-chain joined through a disulfide bond with the single-pass transmembrane β-chain containing the tyrosine kinase domain (Figure 3B) [135,136]. HGF (hepatocyte growth factor), the only known ligand of MET, is also identified as scatter factor for its ability to promote cell migration, besides cell growth.

#### 4.2.1. Direct Effect of Glycosylation on MET Activity

MET contains 11 *N*-glycosylation sites. The inhibition of *N*-glycosylation results in the block of the intracellular transport of MET but, at the same time, in a partial activation of MET signaling in the absence of ligand [16]. Core fucosylationand *Fut8* are important for MET regulation, as suggested by a mice model in which their suppression inhibits liver regeneration and liver carcinogenesis through inhibition of MET (and EGFR) signaling [25,26]. Bisecting GlcNAcon MET glycans enhances HGF-induced cell scattering in HepG2 cells [29]. This effect is remarkable, owing to the general growth suppressing activity of bisecting GlcNAc. Induction of sLe^x^ on MET by expression of sialyltransferase ST3GAL4 enhances MET signaling and invasion of gastric cancer cells [59], while ST6GAL1 deficiency causes a reduction of α2,6-sialylation of MET and consequently abolishes motility of HCT116 cells [44]. Modulation of *O*-glycosylation in gastric cancer cells by downregulation of GALNT2 (Table 1) causes increased expression and phosphorylation of MET, which results in increased invasion in vitro and in vivo [67]. Core 1 galactosyltransferase-1 (C1GALT1), the enzyme elaborating the T antigen (Table 1), enhances tumor growth by increasing MET dimerization and activation [69].

#### 4.2.2. Effect of Ganglioside Binding on MET Activity

Regulation of MET activity by gangliosides is strongly dependent on their carbohydrate chains. In fact, ganglioside GM3 promotes HGF-stimulated motility of hepatocarcinoma cells via PI3K/Akt signaling, while it inhibits EGF-stimulated motility in the same cells [73]. In breast cancer cells, the disialoganglioside GD2 activates MET in the absence of HGF [87,88,137], leading to metastasis competence, stem cell like properties and activation of epithelial to mesenchymal transition (EMT) [89]. EMT is a crucial process in embryonic development, tissue repair and metastasis through which epithelial cells acquire a mesenchymal-like phenotype, consisting of reduced cell adhesion and increased motility. In microdomains (Figure 2), the ganglioside GM2, but not GM3 or globoside, forms a complex with tetraspanin CD82, which inhibits HGF-induced activation of MET tyrosine kinase and its crosstalk with integrins [85]. Ganglioside GD1a negatively regulates HGF expression through caveolin-1 in murine osteosarcoma cells [92]. However, in canine kidney MDCK cells the highly similar ganglioside GD1α (the structures of the two gangliosides differ only for the position and the type of linkage of a sialic acid residue, Table 1), synthesized by sialyltransferase ST6GALNAC5, results in MET overexpression and phosphorylation and anchorage-independent growth [93].

### 4.3. Receptors of the Vascular Endothelial Growth Factors

The vascular endothelial growth factors (VEGFs) and their RTKs (VEGFRs) constitute a major signaling system, which is necessary for the formation of the circulatory system (vasculogenesis), the growth of new blood vessels from those pre-existing (angiogenesis) and is crucial for cancer progression and metastasis formation. Among the three VEGFR members, only VEGFR2 is able to bind all types of VEGF [138]. VEGF-A is the target of the monoclonal antibody Bevacizumab, in clinical use for the treatment of several advanced cancers.

#### 4.3.1. Direct Effect of Glycosylation on VEGFR Activity

The number of *N*-glycosylation sites of VEGFR1-3 is 13, 18 and 12, respectively. VEGFRs provide multiple examples of direct and indirect modulation by carbohydrates. The importance of *N*-glycosylation on VEGF activity is supported by the observation that treatment of endothelial cells with a glucose analogue thatinterferes with *N*-glycosylation prevents the formation of new capillaries due to the inhibition of Akt and extracellular signal-regulated kinase (ERK) signaling downstream to VEGFR2 [17]. Mice KO for the core fucose synthesizing enzyme *Fut8* display an emphysema-like phenotype. Among the possible explanations, one is based on the finding that *Fut8* is required for VEGFR2 expression. In its absence, the pro-apoptotic factor ceramide is overexpressed, leading to increased apoptosis among the cells of the alveolar septa of the lungs [27].

#### 4.3.2. Effect of Galectin Binding on VEGFR Activity

Both tumor and stromal cells may be responsible for the presence of galectins in the tumor microenvironment [139]. Regardless the origin, galectins play a crucial role in neoangiogenesis and tumor promotion. Two glycan modifications decorating VEGFR2, namely α2,6-sialylation and β1,6-branching, can alternatively regulate the activation of VEGF signaling in the absence of VEGF, a phenomenon at the basis of Bevacizumab resistance. Resistant tumors secrete higher amounts of galectin-1 and express on VEGFR2 β1,6-branched *N*-glycans, synthesized by MGAT5. Binding of galectin-1 to these glycans promotes clustering of the receptors, triggering VEGF-independent activation. On the contrary, the high α2,6-sialylation of Bevacizumab-sensitive cells inhibits galectin-1 binding, maintaining the ligand requirement for receptor activation and Bevacizumab sensitivity [40,140]. Also galectin-3 contributes to activation of VEGFR2 by binding to its β1,6-branched glycans resulting in its plasma membrane retention [41]. Galectins-1 and -3 induce angiogenesis in different endothelial cell lines. While galectin-1 or -3 alone induce only the phosphorylation of VEGFR2, the presence of both galectins is required to induce the phosphorylation of both VEGFR1 and -2 [41,141,142]. An inhibitory effect is played by galectin-9, which prevents the phosphorylation of VEGFR3 (and IGF1R), resulting in inhibition of gastric cancer cells proliferation [143]. Galectins can modulate VEGFR activity also indirectly by interacting with VEGFR-associated molecules. A first example is provided by neuropilin-1, which acts as a co-receptor of VEGFR for VEGF in endothelial cells [144]. Through binding to neuropilin-1, but not to VEGFRs, galectin-1 activates VEGFR2 signaling, resulting in stimulation of the MAPK serine/threonine-protein kinase-1 (SAPK1)/c-Jun *N*-terminal kinase (JNK) and enhanced proliferation and migration of vascular endothelial cells [145]. In addition, galectin-1 bound to neuropilin-1 promotes vascular permeability in complex with VEGFR1 [146]. Pathological lymphangiogenesis (the sprouting of lymphatic vessels from pre-existing lymphatic vessels) is stimulated by galectin-8 through a crosstalk between podoplanin (a heavily glycosylated mucin-type glycoprotein) and integrin-associated VEGFR3 [147]. While in several biological systems the effect of galectin-1 is to promote cancer growth, in the trophoblast tumor cells BeWo it inhibits proliferation. This is due to the fact that of the three RTKs out of 71 modulated by galectin-1 treatment, the signaling of RET and JAK2 was inhibited, while that of VEGFR3 was stimulated [148].

#### 4.3.3. Effect of Ganglioside Binding on VEGFR Activity

Interaction with glycolipids plays an important role in the regulation of VEGF system. Lactosylceramide, but not other related compounds, stimulates VEGF-induced angiogenesis [149], as observed after inhibition of the lactosylceramide synthase B4GALT5 [72]. On the contrary, ganglioside GM3 inhibits the VEGFR2 phosphorylation induced by VEGF and complex gangliosides as GD1a [82]. The mechanism involves both the ligand-receptor binding and the receptor dimerization [81]. Moreover, gangliosides, which are frequently shed by cancer cells in the microenvironment and in body fluids, mediate the activation of VEGFR, by decreasing its activation threshold [150].

### 4.4. Receptors for Fibroblast Growth Factors

Fibroblast growth factor receptors (FGFRs) form a family of four RTKs (FGFR1-4) thatbind to 18 fibroblast growth factors (FGFs), expressed in nearly all tissues, playing important roles during embryonic development and tissue repair. FGF2 is also known as basic fibroblast growth factor (bFGF). FGFs bind to heparan sulfate proteoglycans (HSPG), forming a ternary complex with FGFRs on the cell surface. The inactive FGFR monomer undergoes dimerization and trans-autophosphorylation of several specific tyrosine residues upon binding of two FGF molecules linked to HSPGs. The main intracellular signaling pathways activated through stimulation of FGFR are RAS-MAPK kinase, PI3K-Akt, phospholipase Cγ and STAT [151]. Achondroplasia, a common form of dwarfism, is due to gain of function mutations of *FGFR3* gene, leading to receptor over-activation.

#### 4.4.1. Direct Effect of Glycosylation on FGFR Activity

The number of *N*-glycosylation sites on FGFR1-4 is 8, 8, 6 and 5, respectively. Several studies highlight the role of *N*-glycosylation in the regulation of FGFR activity. The lack of *N*-glycans on FGFR may increase the assembly of the FGF-FGFR-HSPG complex, leading to over-activation [22]. An Asn328Ile point mutation in FGFR has been associated to hypochondroplasia due to the lack of glycosylation, rather than to the aminoacid substitution *per se* [152]. However, inhibition of the enzyme mannose phosphate isomerase, involved in the initial steps of *N*-glycan biosynthesis, indicates that *N*-glycosylation of FGFR is required for signaling [18].

Among the specific carbohydrate determinants regulating FGFR activity, in sperm the presence of the Le^y^ antigen on polylactosaminic chains inhibits FGFR signaling [57]. The role of *O*-glycosylation on FGFR signaling is shown by the effects of inhibition of GALNT3, which increases FGF23 cleavage [153] and by galactosylation of *O*-glycans of FGFR2 due to overexpression of C1GALT1 (Table 1), which results in the activation of the receptor and exacerbation of the malignant phenotype of CRC cells [70].

#### 4.4.2. Effect of Galectin and Ganglioside Binding on FGFR Activity

The integrin-dependent activation of FGFR (and VEGFR) can be mediated also by the clustering operated by the binding of galectin-3 to the β1,6-branched *N*-linked chains of integrins [142]. FGFRs colocalize with gangliosides, tetraspanins, and integrins in ganglioside-enriched microdomains (Figure 2). In human embryonal fibroblasts, integrin activation, following adhesion to ECM substrates, induces FGFR activation and cell proliferation even in the absence of FGF. This integrin–FGFR interaction is strongly inhibited by GM3 and/or tetraspanins [83].

#### 4.4.3. Effect of PolysialylatedN-CAM on FGFR Activity

The adhesion properties of the neural cell adhesion molecule (N-CAM) are regulated by the length of the polysialic acid chains decorating its *N*-linked chains. Polysialylated N-CAM and HSPG share a polyanionic nature. This could explain the fact that FGFR signaling can be activated also by interaction with polysialylated N-CAM, which has been shown to interact directly with FGFR [63] and with FGF2 [154]. In particular, the migration of cells expressing polysialylated N-CAM on ECM was paralleled by activation of the FGFR and its downstream signaling components [155].

### 4.5. Transforming Growth Factor-β Receptors

The transforming growth factor-β (TGFB) family of cytokines comprises four members (TGFB1-4) with complex and double edge biological effects, mediating immune-suppression as well as tissue repair [156]. In cancer cells, TGFB mediates growth arrest but it can also promote tumor growth by inducing a tolerogenic microenvironment and EMT [157]. The TGFB receptors (TGFBRs) are homo- or heterodimeric Ser/Thr kinase receptors grouped in three types (TGFBR1-3), which signal mainly through the SMAD pathway.

#### 4.5.1. Direct Effect of Glycosylation on TGFRB Activity

The number of *N*-glycosylation sites present on TGFBR1-3 is 1, 3 and 5. TGFBR1 completely lacking *N*-glycosylation, after treatment with glycosylation inhibitors or mutation of *N*-glycosylation sites, accumulates intracellularly, with consequent block of the TGFB signaling [19]. The **core** fucosylation is required for TGFB1 function [158] as shown by data from *Fut8* KO mice, whose emphysema-like phenotype can be explained by impaired TGFB signaling [28]. Moreover, lack of core fucosylation attenuates the EMT of cultured human renal tubular cells [159,160,161] and the vascular calcification in a model of uremia [161]. The Lewis antigens sLe^x^ and sLe^a^, products of fucosyltransferases FUT6 and FUT3 respectively (Table 1), expressed on TGFBR1, are necessary to induce EMT, in particular in colon cancer cells [60], while Le^y^ expression on TGFBR enhances SMAD signaling [56,162]. Sialylation and microsatellite instability are associated in CRC. Because of inherited or sporadic inactivating mutations of DNA repair enzymes (mainly *MSH2* and/or *MLH1*, involved in mismatch repair), about 15% of CRC shows the instability of short repeated sequences (microsatellites), acquiring the microsatellite-instable (MSI) phenotype. This phenotype is associated with high frequency of frameshift mutations in genes expressing microsatellite sequences in their coding regions. The TGFBR2 genes possess a stretch of 10 adenine residues, which frequently undergo frameshift mutations in MSI CRC, leading to TGFBR2 inactivation, a crucial mutational step for CRC progression. In the CRC cell line HCT116 expressing the biallelic inactivation of TGFBR2, the reconstitution with a wild type TGFBR2 gene leads to altered sialylation of a variety of glycoproteins [163,164], without any change in the expression of sialyltransferases [164], suggesting a relationship between sialylation and a classical mutational step in CRC. β1,6-branching is involved in the pathogenesis of liver disease through TGFB signaling. Steatohepatitis is a liver disease in which the accumulation of triglycerides in hepatocytes (steatosis) is accompanied by a chronic inflammatory condition that ultimately leads to liver fibrosis (cirrhosis). In the hepatic stellate cells (a type of collagen producing cells involved in liver fibrosis) of a murine model of steatohepatitis, *Mgat5* expression enhances TGFB signaling although it inhibits collagen production, fibrosis, lymphocyte infiltration and progression to steatohepatitis [42].

#### 4.5.2. Glycosylation as Inducer and Product of EMT

**Figure 4:**
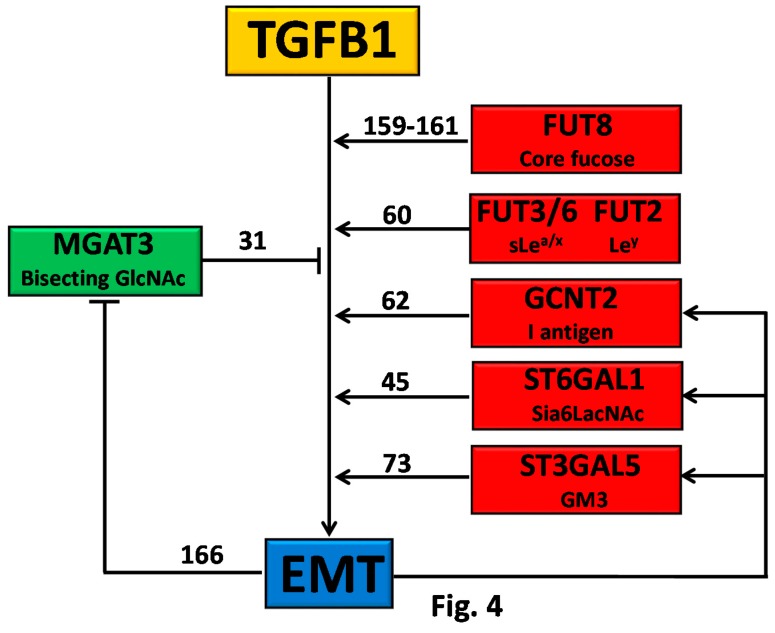
TGFB1 and EMT.Owing to the overexpression of their cognate glycosyltransferases, some carbohydrate structures, indicated on the right, stimulate EMT. In turn, EMT stimulates the glycosyltransferases responsible for the biosynthesis of EMT-promoting structures, originating a self-fueling loop. MGAT3 and its cognate bisecting GlcNAc structure inhibit EMT, however, which inhibits MGAT3 expression, removing a brake to EMT. Numbers indicate the appropriate references.

In some circumstances, self-fueling loops may be generated when a glycosyltransferase product promotes EMT, which, in turn, stimulates the expression of that glycosyltransferase (Figure 4). Examples of self-fueling loops are provided by the *N*-acetylglucosaminyl transferase 2 (GCNT2), which synthesizes I-branched polylactosaminic chains (I blood group antigen) (Table 1) [62], by α2,6 sialylation mediated by ST6GAL1 [45] and by GM3 synthase (ST3GAL5) and GM3 ganglioside [74]. A more complex relationship involves *MGAT3* and its cognate bisecting GlcNAc, which are negatively associated to EMT by modification of E-cadherin [165]. In addition, bisecting GlcNAc synthesized by MGAT3 inhibits TGFB1-induced EMT [31] and is inhibited by TGFB1 [166]. Thus, TGFB1 can remove the brake to EMT represented by bisecting GlcNAc/MGAT3 (Figure 4). 

### 4.6. Insulin Receptor and Insulin-Like Growth Factor Receptors

The insulin receptor (INSR) is a RTK thatcan bind insulin and insulin like growth factor-1 and -2 (IGF-1 and IGF-2). It is composed of two identical chains, each formed by a α and a β chain linked through a cysteine bond; both are the product of alternative splicing and post-translational events from the same transcript of the *INSR* gene. Dysregulation of insulin system leads to diabetes. IGF-1 and -2 are powerful inducers of tissue and body growth, downstream to the growth hormone pathway, and with an important role in cancer growth [167]. The insulin-like growth factor receptors -1 and -2 (IGF1R and IGF2R) are RTKs structurally similar to INSR.

#### 4.6.1. Direct Effect of Glycosylation on INS/IGFR Activity

INSR contains 18 *N*-glycosylation sites, while IGF1R and IGF2R contain 16 and 18 *N*-glycosylation sites, respectively. Tunicamycin inhibition of *N*-glycosylation results in reduced levels of IGF1R because of impaired intracellular transport and processing [15,168]. A close crosstalk exists between IGF1R and androgen receptor (AR), a transcription factor acting as a driver in prostate cancer. AR stimulation increases IGF1R expression in prostate cancer cells [169] and hexosamine production, resulting in increased *N*- and *O*-glycosylation [168]. In turn, *N*-glycosylation of IGF1R is necessary for its plasma membrane localization and full receptor activity [168]. In prostate cancer cells, androgen receptor and IGF1R form a feedback loop in which AR activates IGF1R [169], which, in turn, stimulates AR activity [168,170]. A strong relationship between INSR and IGF1R glycosylation and sexual hormones is also suggested by the marked changes of IGF1R *N*-glycosylation observed in pregnancy [171].

Among specific carbohydrate determinants, the relevance of Lewis antigens is indicated by the fact that FUT7-mediated expression of sLe^x^ on the α subunit of INSR in hepatocarcinoma cells is related to increased signaling [61], while in ovarian cancer the expression of Le^y^ antigen is related to increased expression of IGF1R [172]. The importance of sialylation is revealed by the fact that desialylation of both INSR and IGF1R by NEU1 results in marked effects on cell proliferation in response to insulin treatment [173]. *O*-glycosylation mediated by GALNT2 overexpression (Table 1) attenuates the neoplastic phenotype in neuroblastoma by inhibiting IGF1R dimerization and signaling [68].

#### 4.6.2. Interaction with E-Cadherin

MGAT3 and bisecting GlcNAc play an important role in INSR/IGFR signaling through interaction with E-cadherin. In fact, exogenous E-cadherin administration inhibits insulin signaling, while stimulation with insulin or IGF-1 of cells overexpressing E-cadherin induces downregulation of glycans carrying bisecting glycans, altered intracellular localization of E-cadherin and the acquisition of a mesenchymal-like phenotype [32,33].

#### 4.6.3. Effect of Ganglioside Binding on FGFR Activity

One of the most clinically relevant consequences of diabetes is impaired wound healing. In mouse models of diabetes, the KO of GM3 synthase (ST3GAL1) prevents the diabetes-associated inhibition of wound healing [84]. In fact, proliferation and migration of keratinocytes and activation of IGF1R are suppressed by excess glucose in wild-type cells, but increased in GM3 KO mice, indicating that GM3 inhibits IGF1 signaling [84].

### 4.7. Glucagon Receptor

Insulin and glucagon play opposite roles in glucose homeostasis. The glucagon receptor (GCGR) is a member of the class B G-protein coupled receptors, whose stimulation results in adenylate cyclase activation [174]. It contains 4 *N*-glycosylation sites.

#### Regulation of GCGR Activity by Interaction of β1,6-Branching with Galectin-9

In *Mgat5* KO mice, the glucagon response is reduced, while insulin sensitivity is increased. The activity of Mgat5 and a high concentration of its donor substrate UDP-GlcNAc (which depends on the glucose level) increase the β1,6-branching of *N*-glycans of glucagon receptor. This leads to increased galectin-9 binding and glucagon receptor signaling [43], suggesting that, through the β1,6 branching of glucagon receptor, the glucose level regulates its own homeostasis [43].

### 4.8. Tumor Necrosis Factor Receptors

Both the tumor necrosis factor receptor-1 and -2 (TNFRSF1A and TNFRSF1B), members of the wide tumor necrosis factor receptor superfamily (TNFRSF), can bind tumor necrosis factor-α (TNFA). Upon binding to TNFA, receptors undergo trimerization and interact through their death domain with intracellular adaptor proteins, such as TRAF, activating several downstream signaling pathways related to inflammatory response (NF-kB), stress response (JNK) and apoptotic death (caspase-8) [175].

#### Direct Effect of Glycosylation on TNFRSF Activity

TNFRSF1A and TNFRSF1B contain 3 and 2 *N*-glycosylation sites, respectively. Inhibition of *N*-glycosylation of TNFRSF1A in microglial cells results in inhibition of ligand binding [20]. In macrophages, α2,6-sialylation of TNFRSF1A protects from apoptosis [49]. In Schwann cells stimulated by TNF, galactosyltransferase B4GALT1 triggers an autocrine loop in which the level of B4GALT1 expression regulates MAPK activation, the release of inflammatory mediators and apoptosis [176,177].

Finally, a very peculiar type of glycosylation, the addition of GlcNAc to arginine of the death domains of TNFRSF1A, TRADD and adaptor proteins, is induced by a pathogenic bacterium. This modification disrupts the TNFA signaling, inhibiting apoptosis and necroptosis (a type of regulated necrotic cell death), and is a part of the bacterial defense against the host immune system [178].

## 5. Concluding Remarks

In this review, we have shown that glycosylation can precisely modulate the activity of growth and death factor receptors by different mechanisms, involving the direct effect on receptor transport, ligand binding, dimerization, and signaling. Glycosylation can also affect receptor activity by binding to galectins or by interaction with glycolipids. It appears that most of the glycan structures can differentially promote or inhibit receptor activity depending on the type of receptor and the cellular context (Table 1). While some glyco-epitopes, including the β1,6-branching, the core fucose and diasialogangliosides, appear to exert an activating role in all the systems investigated, other carbohydrate structures (such as Sia6LacNAc and GM3) exert opposite effects on different receptors (Table 1). It can be hypothesized that the effect of the “pan-activatory”glyco-epitopes is related to a common intrinsic nature of the receptors, while the effect of other epitopes remains receptor-specific. The growth of cancer cells is sustained by an extremely complex network of signal transduction pathways often generated at the level of membrane receptors. In this light, the effect of the overexpression of a glycosyltransferase and of its cognate glyco-epitope on cancer growth can be strongly dependent on the receptor expression pattern, resulting in opposite effects in different cancers. The modulation by glycosylation of membrane receptor activity and cell signaling provides a paradigmatic example of how a post-translational modification can generate a flow of information from the cell membrane to the nucleus (outside-in), ultimately regulating gene expression. In some cases, this can originate self-fueling loops. Some membrane receptors or their ligands are the target of molecular drugs aimed at the inhibition of their activity; more effective next generation drugs will be likely produced in the near future. Owing to the crucial role played by glycosylation in the regulation of receptor activity, the development of drugs targeting glyco-epitopes should conceivably be considered as a therapeutically interesting strategy.

## Figures and Tables

**Table 1:** Glycan structures involved in the regulation of receptor activity.

Carbohydrate Determinant	Structure	Glycan Type	Enzyme Activity	Enzyme Abbreviations	Gene Name	Positively Regulated Receptors	Negatively Regulated Receptors
Whole *N*-glycosylation	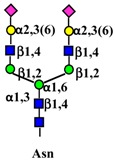	*N*-linked				ERBB1-4 [12,13,14,15] MET [16] VEGFR [17] FGFR [18] TGFBR [19] INSR/IGF1R [15] TNFRSF1A-1B A [20]	ERBB1-4 [21] MET [16] FGFR [22]
Core fucose	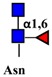	*N*-linked	α1,6 fucosyltransferase 8	FucT-VIII	*FUT8*	ERBB1-4 [23,24,25] MET [25,26] VEGFR [27] TGFBR [28]	
Bisecting GlcNAc	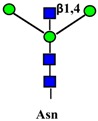	*N*-linked	*N*-acetylglucosaminyl-transferase-III	GnT-III	*MGAT3*	MET [29]	ERBB1-4 [30] TGFBR [31] INSR/IGF1R [32,33]
β1,6 branch	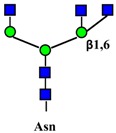	*N*-linked	*N*-acetylglucosaminyl-transferase-V	GnT-V	*MGAT5*	ERBB1-4 [34,35,36,37,38,39] VEGFR [40,41] TGFBR [42] GCGR [43]	
α2,6-sialylated lactosamine Sia6LacNAc	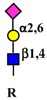	Mainly *N*-linked	α2,6 sialyltransferase 1	α2,6ST, SiaT1	*ST6GAL1*	MET [44] TGFBR [45]	ERBB1-4 [46,47,48] VEGFR [40] TNFRS1A-1B [49]
Lewis^y^	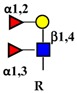	Both *N*- and *O*-linked	α1,2 fucosyltransferase 2α1,2 fucosyltransferase 4	FucT-II FucT-IV	*FUT2* *FUT4*	ERBB1-4 [50,51,52,53,54,55] TGFBR [56] INSR/IGF1R [50]	VEGFR [57]
Sialyl-Lewis^a^	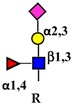	Both *N*- and *O*-linked	α1,4 fucosyltransferase 3	FucT-III	*FUT3*	ERBB1-4 [58]	
Sialyl-Lewis^x^	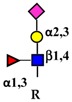	Both *N*- and *O*-linked	α1,4 fucosyltransferase 3, 5,6,7	FucT-III FucT-V FucT-VI FucT-VII	*FUT3* *FUT5* *FUT6* *FUT7*	MET [59] TGFBR [60] INSR/IGF1R [61]	ERBB1-4 [46]
I antigen	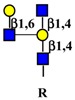	Both *N*- and *O*-linked	*N*-acetylglucosaminyl-transferase 2, branching enzyme	GCNT2	*GCNT2*	TGFBR [62]	
Polysialic acid	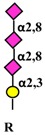	*N*-linked	α2,8 polysialyltransferase 2α2,8 polysialyltransferase 4, PST	STX PST	*ST8SIA2* *ST8SIA4*	FGFR [63]	
GalNAc-Ser/Thr(Tn antigen)	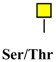	*O*-linked	peptide:*N*-acetylgalactosaminyl- transferases 1-20	GALNT1- GALNT20	*GALNT1-GALNT20*	ERBB1-4 [64,65]	ERBB1-4 [66] MET [67] INSR/IGF1R [68]
Galβ1,3GalNAc-Ser/Thr (T-antigen)	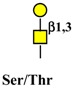	*O*-linked	Core 1 galactosyl-transferase 1, T synthase	C1GALT1	*C1GALT1*	MET [69] VEGFR [70]	
Siaα2,3Galβ1,3GalNAc-Ser/Thr (sialylT-antigen)	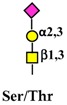	*O*-linked	α2,3 sialyltransferase 1	ST3GAL1	*ST3GAL1*	ERBB1-4 [71]	
Lactosylceramide	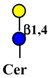	Glycolipid	β1,4 galactosyltransferase 5	B4GALT5	*B4GALT5*	VEGFR [72]	
GM3	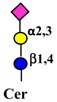	Glycolipid	α2,3 sialyltransferase 5,GM3 synthase	ST3GAL5	*ST3GAL5*	MET [73] TGFBR [74]	ERBB1-4 [73,75,76,77,78,79,80] VEGFR [81,82,83] INSR/IGF1R [84]
GM2	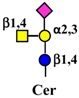	Glycolipid	β1,4 *N*-acetylgalactosaminyl-tranferase 1	B4GALNT1	*B4GALNT1*		MET [85]
GD2/GD3	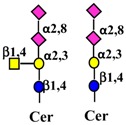	Glycolipid	α2,8 sialyltransferase 1, GD3 synthaseβ1,4 *N*-acetylgalactosaminyl-transferase 1, GD2 synthase	ST8SIA1 B4GALNT1	*ST8SIA1* *B4GALNT1*	ERBB1-4 [86] MET [87,88,89]	
GD1a	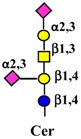	Glycolipid	α2,3 sialyltransferase 2	ST3GalA.2, SAT4, SiaT4b	*ST3GAL2*	ERBB1-4 [75,90,91]	MET [92]
GD1α	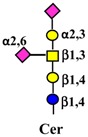	Glycolipid	α2,6 sialyltransferase 5	ST6GALNAC5	*ST6GALNAC5*	MET [93]	

Monosaccharides are represented according to the following code: 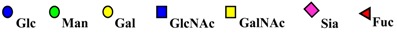. The depicted *N*-linked chain, represents a generic diantennary, di-sialylated *N*-glycan.

## Abbreviations

AKT	protein kinase B
AR	androgen receptor
bFGF	basic growth factor receptor
Cer	ceramide
CRC	colorectal cancer
CSC	cancer stem cells
CTLA-4	cytotoxic T-lymphocyte antigen 4
ECM	extracellular matrix
EGF	epidermal growth factor
EGFR	epidermal growth factor receptor
EMT	epithelial to mesenchymal transition
ER	estrogen receptor
ERK	extracellular signal-regulated kinase
FAK	focal adhesion kinase
FGF	fibroblast growth factor
FGFR	fibroblast growth factor receptor
Fuc	fucose
Gal	galactose
GalNAc	*N*-acetylgalactosamine
GALNT2	*N*-acetylgalactosaminyltransferase 2
Glc	glucose
GlcNAc	*N*-acetylglucosamine
GCGR	glucagon receptor
HGF	hepatocyte growth factor
HSPG	heparan sulfate proteoglycans
IGF-1	insulin-like growth factor-1
IGF1-R	insulin-like growth factor-1 receptor
INSR	insulin receptor
iPSC	induced pluripotent stem cells
JAK	Janus kinase
JNK	c-Jun *N*-terminal
Le^y^	Lewis^y^
Man	man
MAPK	mitogen-activated protein kinase
MSI	microsatellite instability
MUC1	mucin-1
N-CAM	neural cell adhesion molecule
NEU1-4	neuraminidase 1-4
PI3K	phosphatidylinositol-4,5-biphosphate 3-kinase
PIP3	phosphatidylinositol (3,4,5)-triphosphate
PSA	polysialic acid
PST	polysialyltransferase ST8SIA2
RTK	receptor tyrosine kinase
SAPK1	serine/threonine-protein kinase-1
Sia	sialic acid
sT	sialyl-T
Sia6LacNAc	α2,6-sialylated lactosamine
STAT	signal transducer and activator of transcription
STX	polysialyltransferase ST8SIA2
TF	Thomsen–Friedenreich
sLe^a^	sialyl-Lewis^a^
sLe^x^	sialyl-Lewis^x^
TGFB	transforming growth factor-β
TNFA	tumor necrosis factor-α
TNFRSF1A and TNFRSF1B	tumor necrosis factor receptor-1A and -1B
TRADD	TNFRSF1A-associated death domain
TSPAN	tetraspanin
VEGF	vascular endothelial growth factor
VEGFR	vascular endothelial growth factor receptor

## References

[1] Takahashi M., Tsuda T., Ikeda Y., Honke K., Taniguchi N. (2004). Role of *N*-glycans in growth factor signaling. Glycoconj. J..

[2] Takahashi M., Hasegawa Y., Gao C., Kuroki Y., Taniguchi N. (2016). *N*-glycans of growth factor receptors: Their role in receptor function and disease implications. Clin. Sci. Lond..

[3] Takahashi M., Kizuka Y., Ohtsubo K., Gu J., Taniguchi N. (2016). Disease-associated glycans on cell surface proteins. Mol. Asp. Med..

[4] Pakkiriswami S., Couto A., Nagarajan U., Georgiou M. (2016). Glycosylated Notch and Cancer. Front Oncol..

[5] Takeuchi H., Haltiwanger R.S. (2014). Significance of glycosylation in Notch signaling. Biochem. Biophys. Res. Commun..

[6] Tang W., Weng S., Zhang S., Wu W., Dong L., Shen X., Zhang S., Gu J., Xue R. (2013). Direct interaction between surface β1,4-galactosyltransferase 1 and epidermal growth factor receptor (EGFR) inhibits EGFR activation in hepatocellular carcinoma. Biochem. Biophys. Res. Commun..

[7] Kornfeld R., Kornfeld S. (1985). Assembly of asparagine-linked oligosaccharides. Annu. Rev. Biochem..

[8] Bennett E.P., Mandel U., Clausen H., Gerken T.A., Fritz T.A., Tabak L.A. (2012). Control of mucin-type O-glycosylation: A classification of the polypeptide GalNAc-transferase gene family. Glycobiology.

[9] Varki A. (2017). Biological roles of glycans. Glycobiology.

[10] Dall’Olio F., Malagolini N., Trinchera M., Chiricolo M. (2012). Mechanisms of cancer-associated glycosylation changes. Front Biosci..

[11] Pinho S.S., Reis C.A. (2015). Glycosylation in cancer: Mechanisms and clinical implications. Nat. Rev. Cancer.

[12] Tsuda T., Ikeda Y., Taniguchi N. (2000). The Asn-420-linked sugar chain in human epidermal growth factor receptor suppresses ligand-independent spontaneous oligomerization. Possible role of a specific sugar chain in controllable receptor activation. J. Biol. Chem..

[13] Whitson K.B., Whitson S.R., Red-Brewer M.L., McCoy A.J., Vitali A.A., Walker F., Johns T.G., Beth A.H., Staros J.V. (2005). Functional Effects of Glycosylation at Asn-579 of the Epidermal Growth Factor Receptor. Biochemistry.

[14] Yokoe S., Takahashi M., Asahi M., Lee S.H., Li W., Osumi D., Miyoshi E., Taniguchi N. (2007). The Asn418-linked *N*-glycan of ErbB3 plays a crucial role in preventing spontaneous heterodimerization and tumor promotion. Cancer Res..

[15] Contessa J.N., Bhojani M.S., Freeze H.H., Rehemtulla A., Lawrence T.S. (2008). Inhibition of N-linked glycosylation disrupts receptor tyrosine kinase signaling in tumor cells. Cancer Res..

[16] Chen R., Li J., Feng C.H., Chen S.K., Liu Y.P., Duan C.Y., Li H., Xia X.M., He T., Wei M. (2013). c-Met function requires N-linked glycosylation modification of pro-Met. J. Cell Biochem..

[17] Kovacs K., Decatur C., Toro M., Pham D.G., Liu H., Jing Y., Murray T.G., Lampidis T.J., Merchan J.R. (2016). 2-Deoxy-Glucose Downregulates Endothelial AKT and ERK via Interference with N-Linked Glycosylation, Induction of Endoplasmic Reticulum Stress, and GSK3beta Activation. Mol. Cancer Ther..

[18] Cazet A., Charest J., Bennett D.C., Sambrooks C.L., Contessa J.N. (2014). Mannose phosphate isomerase regulates fibroblast growth factor receptor family signaling and glioma radiosensitivity. PLoS ONE.

[19] Kim Y.W., Park J., Lee H.J., Lee S.Y., Kim S.J. (2012). TGF-β sensitivity is determined by N-linked glycosylation of the type II TGF-β receptor. Biochem. J..

[20] Han L., Zhang D., Tao T., Sun X., Liu X., Zhu G., Xu Z., Zhu L., Zhang Y., Liu W. (2015). The role of *N*-Glycan modification of TNFR1 in inflammatory microglia activation. Glycoconj. J..

[21] Ling Y.H., Li T., Perez-Soler R., Haigentz M. (2009). Activation of ER stress and inhibition of EGFR *N*-glycosylation by tunicamycin enhances susceptibility of human non-small cell lung cancer cells to erlotinib. Cancer Chemother. Pharmacol..

[22] Duchesne L., Tissot B., Rudd T.R., Dell A., Fernig D.G. (2006). *N*-glycosylation of fibroblast growth factor receptor 1 regulates ligand and heparan sulfate co-receptor binding. J. Biol. Chem..

[23] Matsumoto K., Yokote H., Arao T., Maegawa M., Tanaka K., Fujita Y., Shimizu C., Hanafusa T., Fujiwara Y., Nishio K. (2008). *N*-Glycan fucosylation of epidermal growth factor receptor modulates receptor activity and sensitivity to epidermal growth factor receptor tyrosine kinase inhibitor. Cancer Sci..

[24] Wang X., Gu J., Ihara H., Miyoshi E., Honke K., Taniguchi N. (2006). Core Fucosylation Regulates Epidermal Growth Factor Receptor-mediated Intracellular Signaling. J. Biol. Chem..

[25] Wang Y., Fukuda T., Isaji T., Lu J., Im S., Hang Q., Gu W., Hou S., Ohtsubo K., Gu J. (2015). Loss of α1,6-fucosyltransferase inhibits chemical-induced hepatocellular carcinoma and tumorigenesis by down-regulating several cell signaling pathways. FASEB J..

[26] Wang Y., Fukuda T., Isaji T., Lu J., Gu W., Lee H.H., Ohkubo Y., Kamada Y., Taniguchi N., Miyoshi E. (2015). Loss of α1,6-fucosyltransferase suppressed liver regeneration: Implication of core fucose in the regulation of growth factor receptor-mediated cellular signaling. Sci. Rep..

[27] Wang X., Fukuda T., Li W., Gao C.X., Kondo A., Matsumoto A., Miyoshi E., Taniguchi N., Gu J. (2009). Requirement of Fut8 for the expression of vascular endothelial growth factor receptor-2: A new mechanism for the emphysema-like changes observed in Fut8-deficient mice. J. Biochem..

[28] Wang X., Inoue S., Gu J., Miyoshi E., Noda K., Li W., Mizuno-Horikawa Y., Nakano M., Asahi M., Takahashi M. (2005). Dysregulation of TGF-β1 receptor activation leads to abnormal lung development and emphysema-like phenotype in core fucose-deficient mice. Proc. Natl. Acad. Sci. USA.

[29] Hyuga M., Hyuga S., Kawasaki N., Ohta M., Itoh S., Niimi S., Kawanishi T., Hayakawa T. (2004). Enhancement of hepatocyte growth factor-induced cell scattering in *N*-acetylglucosaminyltransferase III-transfected HepG2 cells. Biol. Pharm. Bull..

[30] Gu J., Zhao Y., Isaji T., Shibukawa Y., Ihara H., Takahashi M., Ikeda Y., Miyoshi E., Honke K., Taniguchi N. (2004). β1,4-*N*-Acetylglucosaminyltransferase III down-regulates neurite outgrowth induced by costimulation of epidermal growth factor and integrins through the Ras/ERK signaling pathway in PC12 cells. Glycobiology.

[31] Xu Q., Isaji T., Lu Y., Gu W., Kondo M., Fukuda T., Du Y., Gu J. (2012). Roles of *N*-Acetylglucosaminyltransferase III in Epithelial-to-Mesenchymal Transition Induced by Transforming Growth Factor β1 (TGF-β1) in Epithelial Cell Lines. J. Biol. Chem..

[32] De-Freitas-Junior J.C., Carvalho S., Dias A.M., Oliveira P., Cabral J., Seruca R., Oliveira C., Morgado-Diaz J.A., Reis C.A., Pinho S.S. (2013). Insulin/IGF-I signaling pathways enhances tumor cell invasion through bisecting GlcNAc *N*-glycans modulation. an interplay with E-cadherin. PLoS ONE.

[33] De-Freitas-Junior J.C.M., Andrade-da-Costa J., Silva M.C., Pinho S.S. (2017). Glycans as Regulatory Elements of the Insulin/IGF System: Impact in Cancer Progression. Int. J. Mol. Sci..

[34] Liu J., Liu H., Zhang W., Wu Q., Liu W., Liu Y., Pan D., Xu J., Gu J. (2013). *N*-acetylglucosaminyltransferase V confers hepatoma cells with resistance to anoikis through EGFR/PAK1 activation. Glycobiology.

[35] Wang C., Yang Y., Yang Z., Liu M., Li Z., Sun L., Mei C., Chen H., Chen L., Wang L. (2009). EGF-mediated migration signaling activated by *N*-acetylglucosaminyltransferase-V via receptor protein tyrosine phosphatase kappa. Arch. Biochem. Biophys..

[36] Guo H.B., Randolph M., Pierce M. (2007). Inhibition of a specific *N*-glycosylation activity results in attenuation of breast carcinoma cell invasiveness-related phenotypes: Inhibition of epidermal growth factor-induced dephosphorylation of focal adhesion kinase. J. Biol. Chem..

[37] Guo H.B., Johnson H., Randolph M., Lee I., Pierce M. (2009). Knockdown of GnT-Va expression inhibits ligand-induced downregulation of the epidermal growth factor receptor and intracellular signaling by inhibiting receptor endocytosis. Glycobiology.

[38] Guo P., Wang Q.Y., Guo H.B., Shen Z.H., Chen H.L. (2004). *N*-acetylglucosaminyltransferase V modifies the signaling pathway of epidermal growth factor receptor. Cell Mol. Life Sci..

[39] Huang X., Liu T., Wang Q., Zhu W., Meng H., Guo L., Wei T., Zhang J. (2017). Inhibition of *N*-acetylglucosaminyltransferase V enhances the cetuximab-induced radiosensitivity of nasopharyngeal carcinoma cells likely through EGFR *N*-glycan alterations. Glycobiology.

[40] Croci D.O., Cerliani J.P., Dalotto-Moreno T., Mendez-Huergo S.P., Mascanfroni I.D., Dergan-Dylon S., Toscano M.A., Caramelo J.J., Garcia-Vallejo J.J., Ouyang J. (2014). Glycosylation-Dependent Lectin-Receptor Interactions Preserve Angiogenesis in Anti-VEGF Refractory Tumors. Cell.

[41] Markowska A.I., Jefferies K.C., Panjwani N. (2011). Galectin-3 protein modulates cell surface expression and activation of vascular endothelial growth factor receptor 2 in human endothelial cells. J. Biol. Chem..

[42] Kamada Y., Mori K., Matsumoto H., Kiso S., Yoshida Y., Shinzaki S., Hiramatsu N., Ishii M., Moriwaki K., Kawada N. (2012). *N*-Acetylglucosaminyltransferase V regulates TGF-β response in hepatic stellate cells and the progression of steatohepatitis. Glycobiology.

[43] Johswich A., Longuet C., Pawling J., Rahman A.A., Ryczko M., Drucker D.J., Dennis J.W. (2014). *N*-Glycan Remodeling on Glucagon Receptor Is an Effector of Nutrient Sensing by the Hexosamine Biosynthesis Pathway. J. Biol. Chem..

[44] Qian J., Zhu C.H., Tang S., Shen A.J., Ai J., Li J., Geng M.Y., Ding J. (2009). α2,6-hyposialylation of c-Met abolishes cell motility of ST6Gal-I-knockdown HCT116 cells. Acta Pharmacol. Sin..

[45] Lu J., Isaji T., Im S., Fukuda T., Hashii N., Takakura D., Kawasaki N., Gu J. (2014). β-Galactoside α2,6-Sialyltranferase 1 Promotes Transforming Growth Factor-β-mediated Epithelial-Mesenchymal Transition. J. Biol. Chem..

[46] Liu Y.C., Yen H.Y., Chen C.Y., Chen C.H., Cheng P.F., Juan Y.H., Chen C.H., Khoo K.H., Yu C.J., Yang P.C. (2011). Sialylation and fucosylation of epidermal growth factor receptor suppress its dimerization and activation in lung cancer cells. Proc. Natl. Acad. Sci. USA.

[47] Yen H.Y., Liu Y.C., Chen N.Y., Tsai C.F., Wang Y.T., Chen Y.J., Hsu T.L., Yang P.C., Wong C.H. (2015). Effect of sialylation on EGFR phosphorylation and resistance to tyrosine kinase inhibition. Proc. Natl. Acad. Sci. USA.

[48] Park J.J., Yi J.Y., Jin Y.B., Lee Y.J., Lee J.S., Lee Y.S., Ko Y.G., Lee M. (2012). Sialylation of epidermal growth factor receptor regulates receptor activity and chemosensitivity to gefitinib in colon cancer cells. Biochem. Pharmacol..

[49] Liu Z., Swindall A.F., Kesterson R.A., Schoeb T.R., Bullard D.C., Bellis S.L. (2011). ST6Gal-I regulates macrophage apoptosis via α2-6 sialylation of the TNFR1 death receptor. J. Biol. Chem..

[50] Liu J.J., Lin B., Hao Y.Y., Li F.F., Liu D.W., Qi Y., Zhu L.C., Zhang S.L., Iwamori M. (2010). Lewis^y^ antigen stimulates the growth of ovarian cancer cells via regulation of the epidermal growth factor receptor pathway. Oncol. Rep..

[51] Shan X., Aziz F., Tian L.L., Wang X.Q., Yan Q., Liu J.W. (2015). Ginsenoside Rg3-induced EGFR/MAPK pathway deactivation inhibits melanoma cell proliferation by decreasing FUT4/LeY expression. Int. J. Oncol..

[52] Tian L., Shen D., Li X., Shan X., Wang X., Yan Q., Liu J. (2016). Ginsenoside Rg3 inhibits epithelial-mesenchymal transition (EMT) and invasion of lung cancer by down-regulating FUT4. Oncotarget.

[53] Kawai S., Kato S., Imai H., Okada Y., Ishioka C. (2013). Suppression of FUT1 attenuates cell proliferation in the HER2-overexpressing cancer cell line NCI-N87. Oncol. Rep..

[54] Zhang Z., Sun P., Liu J., Fu L., Yan J., Liu Y., Yu L., Wang X., Yan Q. (2008). Suppression of FUT1/FUT4 expression by siRNA inhibits tumor growth. Biochim. Biophys. Acta.

[55] Zheng Q., Cui X., Zhang D., Yang Y., Yan X., Liu M., Niang B., Aziz F., Liu S., Yan Q. (2017). miR-200b inhibits proliferation and metastasis of breast cancer by targeting fucosyltransferase IV and alpha1,3-fucosylated glycans. Oncogenesis.

[56] Li F.F., Liu J.J., Liu D.W., Lin B., Hao Y.Y., Cong J.P., Zhu L.C., Gao S., Zhang S.L., Iwamori M. (2012). Lewis Y regulates signaling molecules of the transforming growth factor beta pathway in ovarian carcinoma-derived RMG-I cells. Int. J. Oncol..

[57] Sugihara K., Shibata T.K., Takata K., Kimura T., Kanayama N., Williams R., Hatakeyama S., Akama T.O., Kuo C.W., Khoo K.H. (2013). Attenuation of fibroblast growth factor signaling by poly-*N*-acetyllactosamine type glycans. FEBS Lett..

[58] Duarte H.O., Balmana M., Mereiter S., Osorio H., Gomes J., Reis C.A. (2017). Gastric Cancer Cell Glycosylation as a Modulator of the ErbB2 Oncogenic Receptor. Int. J. Mol. Sci..

[59] Gomes C., Osorio H., Pinto M.T., Campos D., Oliveira M.J., Reis C.A. (2013). Expression of ST3GAL4 leads to SLe^x^ expression and induces c-Met activation and an invasive phenotype in gastric carcinoma cells. PLoS ONE.

[60] Hirakawa M., Takimoto R., Tamura F., Yoshida M., Ono M., Murase K., Sato Y., Osuga T., Sato T., Iyama S. (2014). Fucosylated TGF-β receptors transduces a signal for epithelial-mesenchymal transition in colorectal cancer cells. Br. J. Cancer.

[61] Wang Q.Y., Zhang Y., Chen H.J., Shen Z.H., Chen H.L. (2007). α1,3-fucosyltransferase-VII regulates the signaling molecules of the insulin receptor pathway. FEBS J..

[62] Zhang H., Meng F., Wu S., Kreike B., Sethi S., Chen W., Miller F.R., Wu G. (2011). Engagement of I-Branching β-1,6-*N*-Acetylglucosaminyltransferase 2 in Breast Cancer Metastasis and TGF-β Signaling. Cancer Res..

[63] Kiselyov V.V., Soroka V., Berezin V., Bock E. (2005). Structural biology of NCAM homophilic binding and activation of FGFR. J. Neurochem..

[64] Lin M.C., Huang M.J., Liu C.H., Yang T.L., Huang M.C. (2014). GALNT2 enhances migration and invasion of oral squamous cell carcinoma by regulating EGFR glycosylation and activity. Oral Oncol..

[65] Lin T.C., Chen S.T., Huang M.C., Huang J., Hsu C.L., Juan H.F., Lin H.H., Chen C.H. (2017). GALNT6 expression enhances aggressive phenotypes of ovarian cancer cells by regulating EGFR activity. Oncotarget..

[66] Wu Y.M., Liu C.H., Hu R.H., Huang M.J., Lee J.J., Chen C.H., Huang J., Lai H.S., Lee P.H., Hsu W.M. (2011). Mucin Glycosylating Enzyme GALNT2 Regulates the Malignant Character of Hepatocellular Carcinoma by Modifying the EGF Receptor. Cancer Res..

[67] Liu S.Y., Shun C.T., Hung K.Y., Juan H.F., Hsu C.L., Huang M.C., Lai I.R. (2016). Mucin glycosylating enzyme GALNT2 suppresses malignancy in gastric adenocarcinoma by reducing MET phosphorylation. Oncotarget.

[68] Ho W.L., Chou C.H., Jeng Y.M., Lu M.Y., Yang Y.L., Jou S.T., Lin D.T., Chang H.H., Lin K.H., Hsu W.M. (2014). GALNT2 suppresses malignant phenotypes through IGF-1 receptor and predicts favorable prognosis in neuroblastoma. Oncotarget.

[69] Wu Y.M., Liu C.H., Huang M.J., Lai H.S., Lee P.H., Hu R.H., Huang M.C. (2013). C1GALT1 enhances proliferation of hepatocellular carcinoma cells via modulating MET glycosylation and dimerization. Cancer Res..

[70] Hung J.S., Huang J., Lin Y.C., Huang M.J., Lee P.H., Lai H.S., Liang J.T., Huang M.C. (2014). C1GALT1 overexpression promotes the invasive behavior of colon cancer cells through modifying O-glycosylation of FGFR2. Oncotarget.

[71] Wen K.C., Sung P.L., Hsieh S.L., Chou Y.T., Lee O.K., Wu C.W., Wang P.H. (2017). α2,3-sialyltransferase type I regulates migration and peritoneal dissemination of ovarian cancer cells. Oncotarget.

[72] Kolmakova A., Rajesh M., Zang D., Pili R., Chatterjee S. (2009). VEGF recruits lactosylceramide to induce endothelial cell adhesion molecule expression and angiogenesis in vitro and in vivo. Glycoconj. J..

[73] Li Y., Huang X., Wang C., Li Y., Luan M., Ma K. (2015). Ganglioside GM3 exerts opposite effects on motility via epidermal growth factor receptor and hepatocyte growth factor receptor-mediated migration signaling. Mol. Med. Rep..

[74] Kim S.J., Chung T.W., Choi H.J., Kwak C.H., Song K.H., Suh S.J., Kwon K.M., Chang Y.C., Park Y.G., Chang H.W. (2013). Ganglioside GM3 participates in the TGF-β1-induced epithelial-mesenchymal transition of human lens epithelial cells. Biochem. J..

[75] Kim S.M., Jung J.U., Ryu J.S., Jin J.W., Yang H.J., Ko K., You H.K., Jung K.Y., Choo Y.K. (2008). Effects of gangliosides on the differentiation of human mesenchymal stem cells into osteoblasts by modulating epidermal growth factor receptors. Biochem. Biophys. Res. Commun..

[76] Guan F., Handa K., Hakomori S.I. (2011). Regulation of epidermal growth factor receptor through interaction of ganglioside GM3 with GlcNAc of N-linked glycan of the receptor: Demonstration in ldlD cells. Neurochem. Res..

[77] Handa K., Hakomori S.I. (2012). Carbohydrate to carbohydrate interaction in development process and cancer progression. Glycoconj. J..

[78] Kawashima N., Yoon S.J., Itoh K., Nakayama K. (2009). Tyrosine kinase activity of epidermal growth factor receptor is regulated by GM3 binding through carbohydrate to carbohydrate interactions. J. Biol. Chem..

[79] Yoon S.J., Nakayama K., Hikita T., Handa K., Hakomori S.I. (2006). Epidermal growth factor receptor tyrosine kinase is modulated by GM3 interaction with N-linked GlcNAc termini of the receptor. Proc. Natl. Acad. Sci. USA.

[80] Daniotti J.L., Crespo P.M., Yamashita T. (2006). In vivo modulation of epidermal growth factor receptor phosphorylation in mice expressing different gangliosides. J. Cell Biochem..

[81] Chung T.W., Kim S.J., Choi H.J., Kim K.J., Kim M.J., Kim S.H., Lee H.J., Ko J.H., Lee Y.C., Suzuki A. (2009). Ganglioside GM3 inhibits VEGF/VEGFR-2-mediated angiogenesis: Direct interaction of GM3 with VEGFR-2. Glycobiology.

[82] Mukherjee P., Faber A.C., Shelton L.M., Baek R.C., Chiles T.C., Seyfried T.N. (2008). Thematic Review Series: Sphingolipids. Ganglioside GM3 suppresses the proangiogenic effects of vascular endothelial growth factor and ganglioside GD1a. J. Lipid Res..

[83] Toledo M.S., Suzuki E., Handa K., Hakomori S. (2005). Effect of ganglioside and tetraspanins in microdomains on interaction of integrins with fibroblast growth factor receptor. J. Biol. Chem..

[84] Wang X.Q., Lee S., Wilson H., Seeger M., Iordanov H., Gatla N., Whittington A., Bach D., Lu J.Y., Paller A.S. (2014). Ganglioside GM3 depletion reverses impaired wound healing in diabetic mice by activating IGF-1 and insulin receptors. J. Investig. Dermatol..

[85] Todeschini A.R., Dos Santos J.N., Handa K., Hakomori S.I. (2007). Ganglioside GM2-tetraspanin CD82 complex inhibits met and its cross-talk with integrins, providing a basis for control of cell motility through glycosynapse. J. Biol. Chem..

[86] Liang Y.J., Wang C.Y., Wang I.A., Chen Y.W., Li L.T., Lin C.Y., Ho M.Y., Chou T.L., Wang Y.H., Chiou S.P. (2017). Interaction of glycosphingolipids GD3 and GD2 with growth factor receptors maintains breast cancer stem cell phenotype. Oncotarget.

[87] Cazet A., Lefebvre J., Adriaenssens E., Julien S., Bobowski M., Grigoriadis A., Tutt A., Tulasne D., Le Bourhis X., Delannoy P. (2010). GD3 synthase expression enhances proliferation and tumor growth of MDA-MB-231 breast cancer cells through c-Met activation. Mol. Cancer Res..

[88] Cazet A., Bobowski M., Rombouts Y., Lefebvre J., Steenackers A., Popa I., Guerardel Y., Le Bourhis X., Tulasne D., Delannoy P. (2012). The ganglioside GD2 induces the constitutive activation of c-Met in MDA-MB-231 breast cancer cells expressing the GD3 synthase. Glycobiology.

[89] Sarkar T.R., Battula V.L., Werden S.J., Vijay G.V., Ramirez-Pena E.Q., Taube J.H., Chang J.T., Miura N., Porter W., Sphyris N. (2015). GD3 synthase regulates epithelial-mesenchymal transition and metastasis in breast cancer. Oncogene.

[90] Liu Y., Li R., Ladisch S. (2004). Exogenous ganglioside GD1a enhances epidermal growth factor receptor binding and dimerization. J. Biol. Chem..

[91] Yang H.J., Jung K.Y., Kwak D.H., Lee S.H., Ryu J.S., Kim J.S., Chang K.T., Lee J.W., Choo Y.K. (2011). Inhibition of ganglioside GD1a synthesis suppresses the differentiation of human mesenchymal stem cells into osteoblasts. Dev. Growth Differ..

[92] Zhang L., Wang Y., Wang L., Cao T., Hyuga S., Sato T., Wu Y., Yamagata S., Yamagata T. (2011). Ganglioside GD1a negatively regulates hepatocyte growth factor expression through caveolin-1 at the transcriptional level in murine osteosarcoma cells. Biochim. Biophys. Acta.

[93] Chu C., Bottaro D.P., Betenbaugh M.J., Shiloach J. (2016). Stable Ectopic Expression of ST6GALNAC5 Induces Autocrine MET Activation and Anchorage-Independence in MDCK Cells. PLoS ONE.

[94] Dall’Olio F., Malagolini N., Trinchera M., Chiricolo M. (2014). Sialosignaling: Sialyltransferases as engines of self-fueling loops in cancer progression. Biochim. Biophys. Acta.

[95] Contessa J.N., Bhojani M.S., Freeze H.H., Ross B.D., Rehemtulla A., Lawrence T.S. (2010). Molecular imaging of N-linked glycosylation suggests glycan biosynthesis is a novel target for cancer therapy. Clin. Cancer Res..

[96] Weston B.W., Hiller K.M., Mayben J.P., Manousos G.A., Bendt K.M., Liu R., Cusack J.C. (1999). Expression of human α1,3 fucosyltransferase antisense sequences inhibits selectin-mediated adhesion and liver metastasis of colon carcinoma cells. Cancer Res..

[97] Rabinovich G.A., Toscano M.A. (2009). Turning ‘sweet’ on immunity: Galectin-glycan interactions in immune tolerance and inflammation. Nat. Rev. Immunol..

[98] Kobayashi K., Morishita A., Iwama H., Fujita K., Okura R., Fujihara S., Yamashita T., Fujimori T., Kato K., Kamada H. (2015). Galectin-9 suppresses cholangiocarcinoma cell proliferation by inducing apoptosis but not cell cycle arrest. Oncol. Rep..

[99] Dennis J.W., Lau K.S., Demetriou M., Nabi I.R. (2009). Adaptive regulation at the cell surface by *N*-glycosylation. Traffic.

[100] Lau K.S., Partridge E.A., Grigorian A., Silvescu C.I., Reinhold V.N., Demetriou M., Dennis J.W. (2007). Complex *N*-glycan number and degree of branching cooperate to regulate cell proliferation and differentiation. Cell.

[101] Mendelsohn R., Cheung P., Berger L., Partridge E., Lau K., Datti A., Pawling J., Dennis J.W. (2007). Complex *N*-glycan and metabolic control in tumor cells. Cancer Res..

[102] Taniguchi N. (2007). A sugar-coated switch for cellular growth and arrest. Nat. Chem. Biol..

[103] Kaucic K., Liu Y., Ladisch S. (2006). Modulation of growth factor signaling by gangliosides: Positive or negative?. Methods Enzymol..

[104] Oblinger J.L., Boardman C.L., Yates A.J., Burry R.W. (2003). Domain-dependent modulation of PDGFRβ by ganglioside GM1. J. Mol. Neurosci..

[105] Le Naour F., Andre M., Boucheix C., Rubinstein E. (2006). Membrane microdomains and proteomics: Lessons from tetraspanin microdomains and comparison with lipid rafts. Proteomics.

[106] Miyagi T., Wada T., Yamaguchi K., Shiozaki K., Sato I., Kakugawa Y., Yamanami H., Fujiya T. (2008). Human sialidase as a cancer marker. Proteomics.

[107] Miyagi T., Takahashi K., Hata K., Shiozaki K., Yamaguchi K. (2012). Sialidase significance for cancer progression. Glycoconj. J..

[108] Linggi B., Carpenter G. (2006). ErbB receptors: New insights on mechanisms and biology. Trends Cell Biol..

[109] Lin S.Y., Makino K., Xia W., Matin A., Wen Y., Kwong K.Y., Bourguignon L., Hung M.C. (2001). Nuclear localization of EGF receptor and its potential new role as a transcription factor. Nat. Cell Biol..

[110] Takahashi M., Yokoe S., Asahi M., Lee S.H., Li W., Osumi D., Miyoshi E., Taniguchi N. (2008). *N*-glycan of ErbB family plays a crucial role in dimer formation and tumor promotion. Biochim. Biophys. Acta.

[111] Minami A., Shimono Y., Mizutani K., Nobutani K., Momose K., Azuma T., Takai Y. (2013). Reduction of the ST6 β-Galactosamide α-2,6-Sialyltransferase 1 (ST6GAL1)-catalyzed Sialylation of Nectin-like Molecule 2/Cell Adhesion Molecule 1 and Enhancement of ErbB2/ErbB3 Signaling by MicroRNA-199a. J. Biol. Chem..

[112] Mozzi A., Forcella M., Riva A., Difrancesco C., Molinari F., Martin V., Papini N., Bernasconi B., Nonnis S., Tedeschi G. (2015). NEU3 activity enhances EGFR activation without affecting EGFR expression and acts on its sialylation levels. Glycobiology.

[113] Wada T., Hata K., Yamaguchi K., Shiozaki K., Koseki K., Moriya S., Miyagi T. (2007). A crucial role of plasma membrane-associated sialidase in the survival of human cancer cells. Oncogene.

[114] Lillehoj E.P., Hyun S.W., Feng C., Zhang L., Liu A., Guang W., Nguyen C., Luzina I.G., Atamas S.P., Passaniti A. (2012). NEU1 sialidase expressed in human airway epithelia regulates epidermal growth factor receptor (EGFR) and MUC1 protein signaling. J. Biol. Chem..

[115] Lajoie P., Partridge E.A., Guay G., Goetz J.G., Pawling J., Lagana A., Joshi B., Dennis J.W., Nabi I.R. (2007). Plasma membrane domain organization regulates EGFR signaling in tumor cells. J. Cell Biol..

[116] Ligtenberg M.J., Kruijshaar L., Buijs F., Van Meijer M., Litvinov S.V., Hilkens J. (1992). Cell-associated episialin is a complex containing two proteins derived from a common precursor. J. Biol. Chem..

[117] De Oliveira J.T., de Matos A.J., Santos A.L., Pinto R., Gomes J., Hespanhol V., Chammas R., Manninen A., Bernardes E.S., Reis C.A. (2011). Sialylation regulates galectin-3/ligand interplay during mammary tumour progression—A case of targeted uncloaking. Int. J. Dev. Biol..

[118] Huang L., Ren J., Chen D., Li Y., Kharbanda S., Kufe D. (2003). MUC1 cytoplasmic domain coactivates Wnt target gene transcription and confers transformation. Cancer Biol. Ther..

[119] Merlin J., Stechly L., De Beauce S., Monte D., Leteurtre E., Van Seuningen I., Huet G., Pigny P. (2011). Galectin-3 regulates MUC1 and EGFR cellular distribution and EGFR downstream pathways in pancreatic cancer cells. Oncogene.

[120] Piyush T., Chacko A.R., Sindrewicz P., Hilkens J., Rhodes J.M., Yu L.G. (2017). Interaction of galectin-3 with MUC1 on cell surface promotes EGFR dimerization and activation in human epithelial cancer cells. Cell Death. Differ..

[121] Weissenbacher T., Vrekoussis T., Roeder D., Makrigiannakis A., Mayr D., Ditsch N., Friese K., Jeschke U., Dian D. (2013). Analysis of Epithelial Growth Factor-Receptor (EGFR) Phosphorylation in Uterine Smooth Muscle Tumors: Correlation to Mucin-1 and Galectin-3 Expression. Int. J. Mol. Sci..

[122] Okita K., Ichisaka T., Yamanaka S. (2007). Generation of germline-competent induced pluripotent stem cells. Nature.

[123] Kuo H.Y., Hsu H.T., Chen Y.C., Chang Y.W., Liu F.T., Wu C.W. (2015). Galectin-3 modulates the EGFR signaling-mediated regulation of Sox2 expression via c-Myc in lung cancer. Glycobiology.

[124] Tsai C.H., Tzeng S.F., Chao T.K., Tsai C.Y., Yang Y.C., Lee M.T., Hwang J.J., Chou Y.C., Tsai M.H., Cha T.L. (2016). Metastatic Progression of Prostate Cancer Is Mediated by Autonomous Binding of Galectin-4-O-Glycan to Cancer Cells. Cancer Res..

[125] Milani S., Sottocornola E., Zava S., Berselli P., Berra B., Colombo I. (2007). Ganglioside GM(3) is stably associated to tyrosine-phosphorylated ErbB2/EGFR receptor complexes and EGFR monomers, but not to ErbB2. Biochim. Biophys. Acta.

[126] Milani S., Sottocornola E., Zava S., Galbiati M., Berra B., Colombo I. (2010). Gangliosides influence EGFR/ErbB2 heterodimer stability but they do not modify EGF-dependent ErbB2 phosphorylation. Biochim. Biophys. Acta.

[127] Sottocornola E., Misasi R., Mattei V., Ciarlo L., Gradini R., Garofalo T., Berra B., Colombo I., Sorice M. (2006). Role of gangliosides in the association of ErbB2 with lipid rafts in mammary epithelial HC11 cells. FEBS J..

[128] Zurita A.R., Crespo P.M., Koritschoner N.P., Daniotti J.L. (2004). Membrane distribution of epidermal growth factor receptors in cells expressing different gangliosides. Eur. J. Biochem..

[129] Odintsova E., Butters T.D., Monti E., Sprong H., van Meer G., Berditchevski F. (2006). Gangliosides play an important role in the organization of CD82-enriched microdomains. Biochem. J..

[130] Wang X.Q., Sun P., Paller A.S. (2005). Gangliosides inhibit urokinase-type plasminogen activator (uPA)-dependent squamous carcinoma cell migration by preventing uPA receptor/αβ integrin/epidermal growth factor receptor interactions. J. Investig. Dermatol..

[131] Wang X.Q., Sun P., Go L., Koti V., Fliman M., Paller A.S. (2006). Ganglioside GM3 Promotes Carcinoma Cell Proliferation via Urokinase Plasminogen Activator-Induced Extracellular Signal-Regulated Kinase-Independent p70S6 Kinase Signaling. J. Investig. Dermatol..

[132] Wang X.Q., Yan Q., Sun P., Liu J.W., Go L., McDaniel S.M., Paller A.S. (2007). Suppression of epidermal growth factor receptor signaling by protein kinase C-alpha activation requires CD82, caveolin-1, and ganglioside. Cancer Res..

[133] Li Z., Zong H., Kong X., Zhang S., Wang H., Sun Q., Gu J. (2006). Cell Surface β1,4-galactosyltransferase 1 promotes apoptosis by inhibiting epidermal growth factor receptor pathway. Mol. Cell Biochem..

[134] Liu Y., Su Y., Wiznitzer M., Epifano O., Ladisch S. (2008). Ganglioside depletion and EGF responses of human GM3 synthase-deficient fibroblasts. Glycobiology.

[135] Organ S.L., Tsao M.S. (2011). An overview of the c-MET signaling pathway. Ther. Adv. Med. Oncol..

[136] Granito A., Guidetti E., Gramantieri L. (2015). c-MET receptor tyrosine kinase as a molecular target in advanced hepatocellular carcinoma. J. Hepatocell. Carcinoma.

[137] Cazet A., Groux-Degroote S., Teylaert B., Kwon K.M., Lehoux S., Slomianny C., Kim C.H., Lehoux S., Delannoy P. (2009). GD3 synthase overexpression enhances proliferation and migration of MDA-MB-231 breast cancer cells. Biol. Chem..

[138] Stuttfeld E., Ballmer-Hofer K. (2009). Structure and function of VEGF receptors. IUBMB. Life.

[139] Tang D., Gao J., Wang S., Ye N., Chong Y., Huang Y., Wang J., Li B., Yin W., Wang D. (2016). Cancer-associated fibroblasts promote angiogenesis in gastric cancer through galectin-1 expression. Tumour. Biol..

[140] Stanley P. (2014). Galectin-1 Pulls the Strings on VEGFR2. Cell.

[141] D’Haene N., Sauvage S., Maris C., Adanja I., Le Mercier M., Decaestecker C., Baum L., Salmon I. (2013). VEGFR1 and VEGFR2 involvement in extracellular galectin-1- and galectin-3-induced angiogenesis. PLoS ONE.

[142] Markowska A.I., Liu F.T., Panjwani N. (2010). Galectin-3 is an important mediator of VEGF- and bFGF-mediated angiogenic response. J. Exp. Med..

[143] Takano J., Morishita A., Fujihara S., Iwama H., Kokado F., Fujikawa K., Fujita K., Chiyo T., Tadokoro T., Sakamoto T. (2016). Galectin-9 suppresses the proliferation of gastric cancer cells in vitro. Oncol. Rep..

[144] Herzog B., Pellet-Many C., Britton G., Hartzoulakis B., Zachary I.C. (2011). VEGF binding to NRP1 is essential for VEGF stimulation of endothelial cell migration, complex formation between NRP1 and VEGFR2, and signaling via FAK Tyr407 phosphorylation. Mol. Biol. Cell.

[145] Hsieh S.H., Ying N.W., Wu M.H., Chiang W.F., Hsu C.L., Wong T.Y., Jin Y.T., Hong T.M., Chen Y.L. (2008). Galectin-1, a novel ligand of neuropilin-1, activates VEGFR-2 signaling and modulates the migration of vascular endothelial cells. Oncogene.

[146] Wu M.H., Ying N.W., Hong T.M., Chiang W.F., Lin Y.T., Chen Y.L. (2014). Galectin-1 induces vascular permeability through the neuropilin-1/vascular endothelial growth factor receptor-1 complex. Angiogenesis.

[147] Chen W.S., Cao Z., Sugaya S., Lopez M.J., Sendra V.G., Laver N., Leffler H., Nilsson U.J., Fu J., Song J. (2016). Pathological lymphangiogenesis is modulated by galectin-8-dependent crosstalk between podoplanin and integrin-associated VEGFR-3. Nat. Commun..

[148] Fischer I., Schulze S., Kuhn C., Friese K., Walzel H., Markert U.R., Jeschke U. (2009). Inhibiton of RET and JAK2 signals and upregulation of VEGFR3 phosphorylation in vitro by galectin-1 in trophoblast tumor cells BeWo. Placenta.

[149] Rajesh M., Kolmakova A., Chatterjee S. (2005). Novel role of lactosylceramide in vascular endothelial growth factor-mediated angiogenesis in human endothelial cells. Circ. Res..

[150] Liu Y., McCarthy J., Ladisch S. (2006). Membrane Ganglioside Enrichment Lowers the Threshold for Vascular Endothelial Cell Angiogenic Signaling. Cancer Res..

[151] Teven C.M., Farina E.M., Rivas J., Reid R.R. (2014). Fibroblast growth factor (FGF) signaling in development and skeletal diseases. Genes Dis..

[152] Winterpacht A., Hilbert K., Stelzer C., Schweikardt T., Decker H., Segerer H., Spranger J., Zabel B. (2000). A novel mutation in FGFR-3 disrupts a putative *N*-glycosylation site and results in hypochondroplasia. Physiol. Genom..

[153] Song L., Linstedt A.D. (2017). Inhibitor of ppGalNAc-T3-mediated O-glycosylation blocks cancer cell invasiveness and lowers FGF23 levels. eLife.

[154] Ono S., Hane M., Kitajima K., Sato C. (2012). Novel regulation of fibroblast growth factor 2 (FGF2)-mediated cell growth by polysialic acid. J. Biol. Chem..

[155] Li J., Dai G., Cheng Y.B., Qi X., Geng M.Y. (2011). Polysialylation promotes neural cell adhesion molecule-mediated cell migration in a fibroblast growth factor receptor-dependent manner, but independent of adhesion capability. Glycobiology.

[156] Massague J. (2012). TGFβ signalling in context. Nat. Rev. Mol. Cell Biol..

[157] Xu J., Lamouille S., Derynck R. (2009). TGF-β-induced epithelial to mesenchymal transition. Cell Res..

[158] Schachter H. (2005). The search for glycan function: Fucosylation of the TGF-β1 receptor is required for receptor activation. Proc. Natl. Acad. Sci. USA.

[159] Lin H., Wang D., Wu T., Dong C., Shen N., Sun Y., Sun Y., Xie H., Wang N., Shan L. (2011). Blocking core fucosylation of TGF-β1 receptors downregulates their functions and attenuates the epithelial-mesenchymal transition of renal tubular cells. Am. J. Physiol. Ren. Physiol..

[160] Venkatachalam M.A., Weinberg J.M. (2013). New wrinkles in old receptors: Core fucosylation is yet another target to inhibit TGF-β signaling. Kidney Int..

[161] Wen X., Liu A., Yu C., Wang L., Zhou M., Wang N., Fang M., Wang W., Lin H. (2016). Inhibiting post-translational core fucosylation prevents vascular calcification in the model of uremia. Int. J. Biochem. Cell Biol..

[162] Li F., Lin B., Hao Y., Li Y., Liu J., Cong J., Zhu L., Liu Q., Zhang S. (2010). Lewis Y Promotes Growth and Adhesion of Ovarian Carcinoma-Derived RMG-I Cells by Upregulating Growth Factors. Int. J. Mol. Sci..

[163] Lee J., Warnken U., Schnolzer M., Gebert J., Kopitz J. (2015). A new method for detection of tumor driver-dependent changes of protein sialylation in a colon cancer cell line reveals nectin-3 as TGFBR2 target. Protein Sci..

[164] Lee J., Ballikaya S., Schonig K., Ball C.R., Glimm H., Kopitz J., Gebert J. (2013). Transforming growth factor beta receptor 2 (TGFBR2) changes sialylation in the microsatellite unstable (MSI) Colorectal cancer cell line HCT116. PLoS ONE.

[165] Pinho S.S., Oliveira P., Cabral J., Carvalho S., Huntsman D., Gartner F., Seruca R., Reis C.A., Oliveira C. (2012). Loss and recovery of Mgat3 and GnT-III Mediated E-cadherin *N*-glycosylation is a mechanism involved in epithelial-mesenchymal-epithelial transitions. PLoS ONE.

[166] Mo C., Liu T., Zhang S., Guo K., Li M., Qin X., Liu Y. (2017). Reduced *N*-acetylglucosaminyltransferase III expression via Smad3 and Erk signaling in TGF-β1-induced HCC EMT model. Discov. Med..

[167] Leroith D., Werner H., Beitner-Johnson D., Roberts C.T. (1995). Molecular and cellular aspects of the insulin-like growth factor I receptor. Endocr. Rev..

[168] Itkonen H.M., Mills I.G. (2013). N-linked glycosylation supports cross-talk between receptor tyrosine kinases and androgen receptor. PLoS ONE.

[169] Pandini G., Mineo R., Frasca F., Roberts C.T., Marcelli M., Vigneri R., Belfiore A. (2005). Androgens up-regulate the insulin-like growth factor-I receptor in prostate cancer cells. Cancer Res..

[170] Sayeed A., Alam N., Trerotola M., Languino L.R. (2012). Insulin-like growth factor 1 stimulation of androgen receptor activity requires β_1A_ integrins. J. Cell. Physiol..

[171] Robajac D., Masnikosa R., Mikovic Z., Nedic O. (2016). Gestation-associated changes in the glycosylation of placental insulin and insulin-like growth factor receptors. Placenta.

[172] Liu D., Liu J., Wang C., Lin B., Liu Q., Hao Y., Zhang S., Iwamori M. (2011). The Stimulation of IGF-1R Expression by Lewis(y) Antigen Provides a Powerful Development Mechanism of Epithelial Ovarian Carcinoma. Int. J. Mol. Sci..

[173] Arabkhari M., Bunda S., Wang Y., Wang A., Pshezhetsky A.V., Hinek A. (2010). Desialylation of insulin receptors and IGF-1 receptors by neuraminidase-1 controls the net proliferative response of L6 myoblasts to insulin. Glycobiology.

[174] Charron M.J., Vuguin P.M. (2015). Lack of glucagon receptor signaling and its implications beyond glucose homeostasis. J. Endocrinol..

[175] Locksley R.M., Killeen N., Lenardo M.J. (2001). The TNF and TNF receptor superfamilies: Integrating mammalian biology. Cell.

[176] Yang H., Yuan Q., Chen Q., Li C., Wu X., Peng C., Kang L., Lu X., Sun H., Zhou Z. (2011). β-1,4-galactosyltransferase I promotes tumor necrosis factor-alpha autocrine via the activation of MAP kinase signal pathways in schwann cells. J. Mol. Neurosci..

[177] Yuan Q., Yang H., Cheng C., Li C., Wu X., Huan W., Sun H., Zhou Z., Wang Y., Zhao Y. (2012). β-1,4-Galactosyltransferase I involved in Schwann cells proliferation and apoptosis induced by tumor necrosis factor-alpha via the activation of MAP kinases signal pathways. Mol. Cell Biochem..

[178] Li S., Zhang L., Yao Q., Li L., Dong N., Rong J., Gao W., Ding X., Sun L., Chen X. (2013). Pathogen blocks host death receptor signalling by arginine GlcNAcylation of death domains. Nature.

